# Resistance Profiles to Second-Line Anti-Tuberculosis Drugs and Their Treatment Outcomes: A Three-Year Retrospective Analysis from South India

**DOI:** 10.3390/medicina59061005

**Published:** 2023-05-23

**Authors:** Radha Gopalaswamy, Nandhini Palani, Dinesh Viswanathan, Bershila Preysingh, Suchithra Rajendran, Vaishnavee Vijayaraghavan, Kannadasan Thangavel, Senthil Devi Vadivel, Hannah Stanley, Kannan Thiruvengadam, Lavanya Jayabal, Kaleeswari Murugesan, Sridhar Rathinam, Asha Frederick, Gomathi Sivaramakrishnan, Chandrasekaran Padmapriyadarsini, Sivakumar Shanmugam

**Affiliations:** 1 ICMR-National Institute for Research in Tuberculosis, Chennai 600031, India; radhagopalaswamy@gmail.com (R.G.);; 2 District TB, Chennai 600056, India; 3 District TB, Kancheepuram 631502, India; 4 GHTM, Tambaram, Chennai 600047, India; 5 State TB, Sathumadural 600006, India

**Keywords:** fluoroquinolone, resistance, treatment regimen, mutations, LPA, NGS

## Abstract

*Background*: Patients with first-line drug resistance (DR) to rifampicin (RIF) or isoniazid (INH) as a first-line (FL) line probe assay (LPA) were subjected to genotypic DST using second-line (SL) LPA to identify SL-DR (including pre-XDR) under the National TB Elimination Program (NTEP), India. SL-DR patients were initiated on different DR-TB treatment regimens and monitored for their outcomes. The objective of this retrospective analysis was to understand the mutation profile and treatment outcomes of SL-DR patients. *Materials and Methods*: A retrospective analysis of mutation profile, treatment regimen, and treatment outcome was performed for SL-DR patients who were tested at ICMR-NIRT, Supra-National Reference Laboratory, Chennai between the years 2018 and 2020. All information, including patient demographics and treatment outcomes, was extracted from the NTEP Ni-kshay database. *Results*: Between 2018 and 2020, 217 patients out of 2557 samples tested were identified with SL-DR by SL-LPA. Among them, 158/217 were FQ-resistant, 34/217 were SLID-resistant, and 25/217 were resistant to both. D94G (Mut3C) of *gyrA* and a1401g of *rrs* were the most predominant mutations in the FQ and SLID resistance types, respectively. Favorable (cured and treatment complete) and unfavorable outcomes (died, lost to follow up, treatment failed, and treatment regimen changed) were recorded in a total of 82/217 and 68/217 patients in the NTEP Ni-kshay database. *Conclusions*: As per the testing algorithm, SL- LPA is used for genotypic DST following identification of first-line resistance, for early detection of SL-DR in India. The fluoroquinolone resistance pattern seen in this study population corelates with the global trend. Early detection of fluoroquinolone resistance and monitoring of treatment outcome can help achieve better patient management.

## 1. Introduction

In 2021, 10.6 million people fell ill with tuberculosis (TB) worldwide, and 63% of the pulmonary TB cases diagnosed were bacteriologically confirmed. From this population, 71% were tested for rifampicin resistance and 6.4% of all rifampicin-resistant cases included multidrug resistant/rifampicin-resistant (MDR/RR) TB, pre-extensively drug-resistant TB (pre-XDR)-TB, and extensively drug-resistant TB (XDR-TB) [1]. Bacteriological confirmation of drug resistance is essential for better patient management, to make the right choice of treatment regimen [1]. In 2020, the World Health Organization (WHO) issued consolidated guidelines for treatment of DR-TB, which include an H mono/poly regimen for isoniazid resistance (without rifampicin resistance) and shorter or longer all-oral regimen containing bedaquiline for MDR/RR-TB [2]. With inclusion of either levofloxacin or moxifloxacin (normal or high dose) in all DR-TB regimens, fluoroquinolone testing is crucial. In 2021, the global coverage of fluoroquinolone testing was 50%, which is very low given the increasing trend of pre-XDR-TB and XDR-TB and poses a risk during the choice of intervention strategy [1]. The line probe assay (LPA) for the second-line (SL) drugs is a rapid molecular test aimed at identifying fluoroquinolone resistance and mutations in specific regions, which corelates with the phenotypic drug susceptibility [3]. In India, there is a well-established guideline for diagnosis and treatment of DR-TB under the National TB Elimination Program (NTEP). The Programmatic Management of Drug resistant TB (PMDT) guideline (2017) covering this study period (2018–2020) was further revised in 2021 with some modification of diagnosis and treatment principles [4,5]. With regard to SL-DR, SL-LPA identified SL-DR as specifically fluoroquinolone (FQ) resistance in the range from 27.4% to 29.6%; second-line injectable drug (SLID) between 1.3 and 1.5% and FQ/SLID resistance in the range 5.3–6.3% among tests performed in India during the years 2018–2020 [6,7,8]. These indicate the good coverage of SL-LPA testing in India as a step towards early detection of DR-TB. In India, an increasing number of reference laboratories for tuberculosis (TB) are becoming certified in FL-LPA and SL-LPA testing, from 56 and 50 laboratories respectively in 2018, to 82 and 72 laboratories respectively in 2023 [9,10]. In line with this increasing SL-LPA testing, some previous studies focused on the mutation profiles of SL-DR [11,12,13,14,15,16,17], but there is paucity of information on the corresponding treatment outcomes of SL-DR patients and trends in resistance mutation. In the present retrospective study spanning three years (2018–2020), it was proposed to look at the mutation profiles and treatment outcomes of SL-DR patients from seven districts of Tamil Nadu and tested using SL-LPA at the Supra National Reference Laboratory (SNRL), Indian Council of Medical Research–National Institute for Research in Tuberculosis (ICMR-NIRT), Chennai, India. The study focus was on the mutation profiles of drug resistance using LPA and the treatment outcomes of SL-DR patients. This retrospective study recommends the rapid optimization of SL-LPA testing among all the reference laboratories under NTEP that perform FL-LPA testing. Additionally, close monitoring of treatment outcomes for SL-DR patients is required, for better patient management.

## 2. Materials and Methods

### 2.1. Study Setting

**Figure 1:**
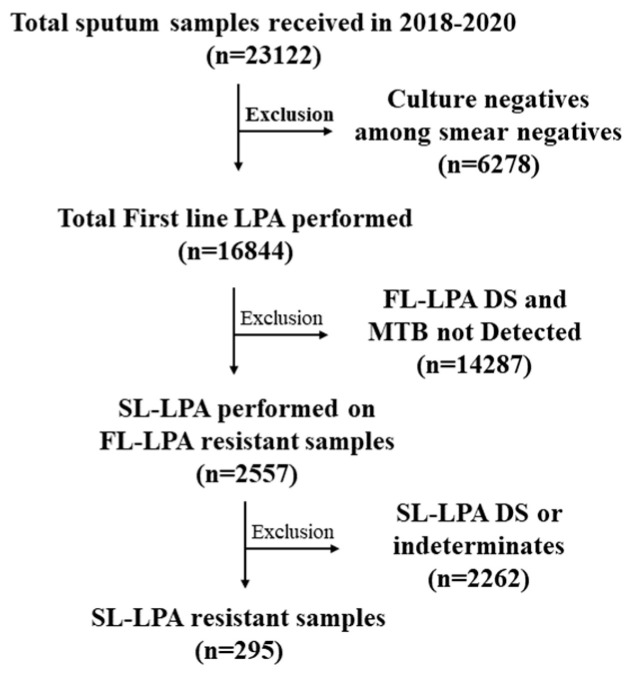
Flowchart of SL-LPA testing for the samples received from 2018 to 2020.

Sputum samples of presumptive DR-TB patients referred from DR-TB centers in Chennai (5 districts), Kancheepuram, and Tiruvallur districts of Tamil Nadu were transported to the SNRL for drug susceptibility testing for first-line and second-line drugs [18]. The current study involved a three year-retrospective analysis (2018–2020) of second-line drug susceptibility testing (DST) as diagnosed by LPA. A total of 23,122 samples were received at ICMR-NIRT during the study period; 16,844 were tested using FL-LPA and 2557 FL-LPA-resistant samples were tested using SL-LPA (Figure 1). The patients were tested for diagnosis and follow-up samples. The results were consolidated for patients whose samples were tested more than once. This study was approved by the ethical committee of ICMR-NIRT (NIRT-IEC:2021-013), Chennai, India. As this study was a retrospective data analysis, a waiver of written informed consent was obtained from NIRT-IEC. Data abstraction was performed until July 2022 from the NTEP Ni-kshay database for any updates of patient status.

### 2.2. Methods

Sputum samples were processed by the standard N-acetyl-L-cysteine and sodium hydroxide (NALC-NaOH) decontamination procedure, as per the standard operating procedure (SOP) in the laboratory [5,18]. A smear was prepared from the deposit, and all smear-positive samples as well as culture positives among smear-negative samples were included in FL-LPA testing. Routinely, culture-negative samples among smear negatives were excluded from FL-LPA testing [5]. DNA was extracted using Genolyse^®^ version 1.0, and FL-LPA was performed using GenoType^®^ MTBDR*plus* version 2.0, as previously described [18]. FL-LPA drug-sensitive (DS) and *M. tuberculosis*-negative samples were excluded from SL-LPA testing. All first-line-resistant patients were included for second-line LPA (SL-LPA) using GenoType^®^ MTBDR*sl* version 2.0 as reflex testing, as per the PMDT 2017 algorithm [4].

Band patterns were analyzed as per the LPA interpretation guidelines [3]. True resistance was identified by the presence of one or more mutation (MUT) probes and the absence of corresponding wild-type (WT) probes, while inferred resistance was identified by the absence of WT probes without the presence of MUT probes. Heteroresistance (co-existence of susceptible and resistant bacteria) was identified by the presence of MUT probes and the corresponding WT probes.

### 2.3. Statistical Analysis

The profile and clinical details of the patients were collected based on the PMDT guidelines and entered into a database with data validation checks. Data were analyzed using STATA version 15.0 (StataCorp, College Station, TX, USA). The data were cross-tabulated by frequency and percentage. The association between the observation year and the result for mutation profile, treatment regimen, and their outcome were tested for significance using cross-tabs and Fisher’s exact test. All the tests were two-tailed and conducted at a significance level of 0.05.

## 3. Results

A total of 2557 samples were tested following detection of first-line resistance during the study period from 2018 to 2020 for second-line (SL) DST using SL- LPA. A total of 295 samples (11.5%) from 217 patients were resistant to second-line drugs (either/both FQs and SLIDs). No discordance in SL-DR was observed between the diagnosis and follow-up samples in the study.

### 3.1. Baseline Characteristics and Resistance Profile

**Figure 2:**
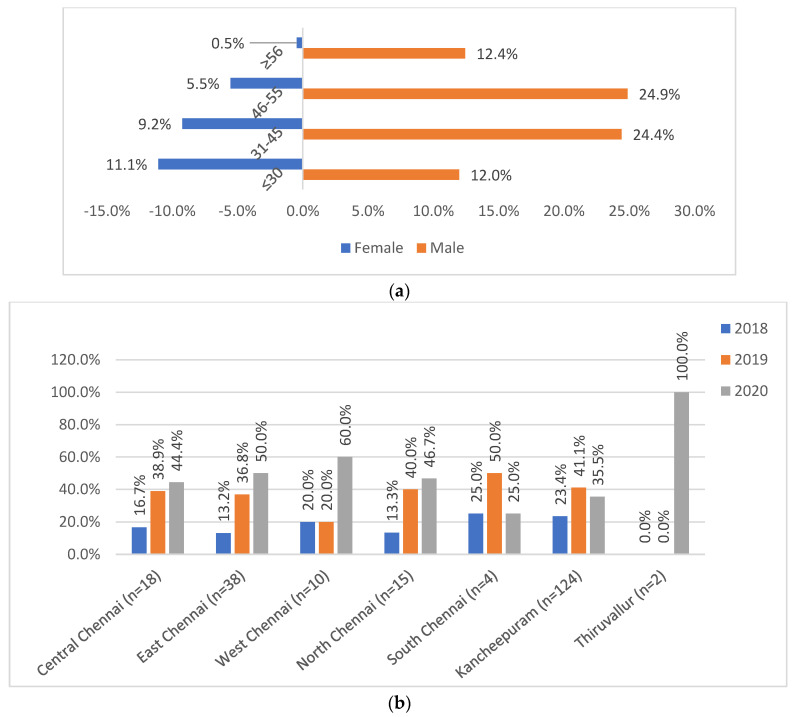
(**a**) Age and gender distribution of patients (*n* = 217). (**b**) District-wise referral of patients (*n* = 217). (**a**) Shows the distribution of male and female gender across the various age groups: ≤30, 31–45, 46–55, and ≥56 years. (**b**) Shows the district-wise (2b) distribution of study patients across districts of Central Chennai, East Chennai, West Chennai, North Chennai, South Chennai, Kancheepuram, and Tiruvallur.

The age, gender, and referrals are given in Figure 2. The 217 SL-DR patients had a median age of 44 years with an interquartile range of 32–51 years. The study had males (73.73%) as the predominant SL-DR population, most commonly in the age groups 31–45 (24.9%) and 46–55 (24.4%). Among them, known HIV-reactive and diabetic cases were 1% and 18%. While 77.4% were new, 16% were previously treated cases and 6% were unclassified. The referrals from Kancheepuram contributed to maximum SL-DR cases (57.14%), followed by Chennai (39.17%), while Tiruvallur (3.7%) was introduced only towards the end of study period (late 2020).

Among the resistance types in patients with SL-DR, FQ resistance (FQ-R) was found in 158/217 cases (72.8%) (Table 1). Resistance to SLID (SLID-R) was found in 34/217 (15.7%) patients. Twenty-five patients (11.5%) presented with resistance to both FQ and SLID. This accounted for 7.18% and 2.30% of FQ-R and SLID-R among all the samples tested during the study period. No significant observations in the trend of SL-DR could be identified over the period of three years (Table 1).

In this study, 2/217 patients had household contacts (HHC) with active disease, which indicated a significant risk of spread of DR-TB. Out of multiple HHC (2–3) screened in each of these two cases, one HHC turned out to be an active TB case and was diagnosed with the same drug-resistant TB as the index case during the same study period. The other case was HHC non-responsive to treatment and consistently positive by smear and culture during the 2 years of ongoing treatment.

### 3.2. Mutation Distribution

SL-LPA identified specific mutations in four genes corresponding to FQ (*gyrA* and *gyrB*), SLID (*rrs*), and low-level kanamycin resistance (*eis*). The distribution of mutations in these four genes among the different patients (numbers reflect more than patient numbers due to inclusion of mutations across FQ-R, SLID-R, as well as FQ/SLID-R patients) during the study period is given in Table 2. Among the FQ-resistant strains, D94G (MUT3C) of *gyrA* (41.0%) was the most common mutation, followed by loss of wild type (codon 536–541) in *gyrB*. For the SLID mutations, a1401g (MUT1) was seen in 61% of patients. D94H (MUT3D) of *gyrA* and E540V (MUT2) of *gyrB* were two mutations that were not observed in this study population. A test of the trend of increasing or decreasing resistance did not show any significance. The majority of the *gyrB* resistance (97%) was inferred due to a lack of wild type, indicating the need to add more mutations in LPA testing for *gyrB*.

The predominant resistance type across three-year period was true resistance for *gyrA* (49.8%), followed by inferred resistance for *gyrB* (14.3%). The inferred resistance, which could be due to lesser-known mutations that could not be detected by this assay, was predominant for *gyrB,* followed by *gyrA*. Heteroresistance (due to mixed infection), identified as the presence of wild types with the presence of respective mutation bands, was observed in all genes (Table 3).

### 3.3. Treatment Regimen

The treatment regimen was changed to a DR-TB regimen based on first-line resistant status, and this information was collected from Ni-kshay database as part of routine NTEP confirmation of regimen change once diagnosed with DR-TB (Table 4). The details of replacement drugs where used were not available. Of 217 SL-DR patients, 20.7% were on an all-oral longer regimen (AOLR) and 12.4% were on a regimen containing newer drugs, including bedaquiline, linezolid, and clofazimine, in addition to SLIDs [4]. Around 11.1% of patients were put on a shorter MDR-TB regimen, while four percent of patients were continued on conventional MDR-TB regimen. An H mono/poly regimen was given to 12% of the patients. In total, 79 (36.4%) of the patients did not have updated treatment regimen details in the database.

### 3.4. Treatment Outcomes

Of the 217 SL-DR patients, 19.4% and 18.4% were cured or treatment-completed, respectively (Table 5). Nearly 14.3% were noted as lost-to-follow up and 4.2% patients were not put on treatment due to patient refusal or other unknown reasons. Meanwhile, 13.8% of the patients with second-line resistance died during the treatment period. Treatment outcome was indicated as failed in 3.2% and regimen changed in 6.0%, with no update on further treatment proceedings. No treatment outcome was assigned for 20.7% of the patients. Overall, favorable (cured and treatment complete) and unfavorable outcomes were recorded in a total of 82/217 and 68/217 patients, respectively.

## 4. Discussion

Diagnosis of DR-TB and intervention strategies to combat it have gained a lot of importance. From central assay to point-of-care diagnosis tests, various kits are currently available and under development for various sample types of pulmonary and extrapulmonary tuberculosis [19]. Along with diagnosis, there needs to be a proper design to intervene and address DR-TB cases. In India, according to the PMDT guidelines, all FL-LPA resistant patients need to be tested using SL-LPA. The Supra-National Reference Laboratory in this retrospective analysis catered to seven districts of Tamil Nadu, including Chennai (5 Districts), Kancheepuram, and later Tiruvallur, during the study period. The SL-DR patients had a median age of 44 years, with a range of 32–51 years and male dominance. Factors associated with treatment outcome such as social status, previous history of TB, comorbidities such as HIV and diabetes, as well as social habits such as drinking, smoking, or tobacco use, did not show any statistical correlation with resistance types. Studies from other parts of India reported an overall frequency of FQ-R, SLID-R, and FQ/SLID-R cases among SL-R patients comparable with this retrospective analysis, with FQ-R always reported as highest among SL-DR patients [11,15,16].

Previous studies indicated a good agreement between molecular (SL-LPA using MTBDR*sl*) and phenotypic tests (LC-DST) for FQ and SLIDs. In some studies, SL-LPA was performed on culture isolates with known DST results for evaluation [20,21]. However, in others studies, patient sputum was used for SL-LPA testing and corelated with phenotypic DST [16,17,22]. Hence, the mutations identified by SL-LPA guided the choice of drug regimen quite well [17,20,21,22]. In the present study, D94G (MUT3C) was the most predominant mutation in *gyrA* for FQ and a1401g in *rrs* for SLID. Across the world, SL-LPA identified these same mutations for FQ and SLID as the most common one. While few studies performed a test on both smear positives and negatives (cultures were raised and used) [12,13,17] as done in this study, others tested with exclusively smear-positive sputum specimens [11,16] or culture isolates [14,15]. Interestingly, in this study, 39.1% of patients had mutations in D94G as well as D94N/Y, which is known to confer high level moxifloxacin resistance [3]. In a previous report from India, studies using MDR-TB culture isolates and mutation profiles in SL-LPA were compared to moxifloxacin susceptibility at 1.0 µg/mL. D94G was shown to be the most prevalent mutation, and selection pressure and transmission was attributed to the accumulation of specific *gyrA* mutations that confer a high level of moxifloxacin resistance [23]. D94G as well as D94N/Y is known to be associated with FQ resistance (both levofloxacin and moxifloxacin) with high confidence, as shown by the WGS catalogue of mutations from India as well as globally [24,25].

The PMDT guidelines (2017) used in India recommended three main DR regimens, which include the conventional MDR-TB regimen, a shorter MDR regimen for MDR/RR-TB, and an H mono/poly regimen for INH resistance without R resistance, all of which include fluoroquinolones and SLIDs [4]. As per the previous guidelines, once identified as SL-DR by SL-LPA or LC-DST, the patient is changed to a regimen for MDR/RR + resistance to FQ/SLI; a regimen for XDR-TB; or a regimen with a newer drug for XDR-TB/failure for MDR/XDR-TB, with inclusion of bedaquiline only where required based on both eligibility and consent. [4]. Both bedaquiline-containing oral regimens were introduced much later: the shorter oral MDRTB regimen in 2018 and AOLR in 2019, which continued to use fluoroquinolones but no longer SLIDs [6,7]. Unfortunately, from the database, there is lack of information for classifying patients identified as shorter MDR in 2018 into either the previous one with SLIDs or the newer oral regimen. With a lack of information in the database on the drugs issued, the patients who were started on bedaquiline treatment could not be clearly delineated. In 2022, the WHO updated its treatment guidelines for DR-TB, whereby MDR/RR-TB patients can be given a new 6-month BPaL or BPaLM regimen, comprising bedaquiline, pretomanid, and linezolid (600 mg) with or without moxifloxacin (for pre-XDR-TB patients with fluoroquinolone resistance) to be used in place of the 9-month or longer (>18 months) regimens [2]. The importance of rapid diagnosis of fluoroquinolone resistance is however crucial, as all these regimens contain either levofloxacin or moxifloxacin (normal or high dose) for all years.

The patients with favorable and unfavorable outcomes across the different regimen types were 37.8% and 31.3%, while 20.7% either had no outcome assigned or were yet to be assigned. Unfavorable outcomes also included lost-to-follow-up, which does not indicate treatment failure, as compared to death or failure or regimen change. Considering the changing treatment regimens with the updating of guidelines and choice of drugs over the study period, it is promising that proper early detection of SL-DR and induction into the appropriate treatment regimen could help increase favorable treatment outcomes. PMDT guidelines recommend capacity building at all NRLs for next generation sequencing (NGS)-based DST. Currently, difficult-to-treat cases are subjected to either WGS or tNGS at a few National Reference Laboratories across the country for rapid procurement of a comprehensive DST profile over a conventional phenotypic DST.

Previous studies from South India have reported less favorable treatment outcomes with newer oral drug regimens for MDR/RR-TB with fluoroquinolone resistance (51%) as compared to MDR/RR-TB (70.6%) [26]. Unfavorable interim treatment outcomes with FQ-R were also reported with the previous conventional MDR/RR-TB regimen in India [27]. Culture conversion rates and/or interim outcomes were good for the bedaquiline-containing regimen across different countries, including India [28]. Interim reports from Korea showed a favorable outcome for newer drug regimens at 12 months in 84.8% of patients [29]. A recent multicenter study including seven countries reported improved sputum conversion in 85% of patients treated with bedaquiline and/or delamanid [30]. Another global cohort study, including 29 countries across different continents showed 88.8% culture conversion (for patients with final outcome declared) and 74.2% favorable treatment outcome with bedaquiline use [31]. However, more detailed reports with final treatment outcome assessments, along with adverse reactions and factors associated with favorable/unfavorable treatment outcomes, are awaited.

PMDT guidelines in India recommended the use of high-dose moxifloxacin as part of AOLR where required [5]. With the use of fluoroquinolones across the different regimens, as levofloxacin or moxifloxacin, it is expected that the use of high-dose moxifloxacin could be appropriate. However, a recent observational cohort study using 600 mg moxifloxacin (high dose) showed that high-dose moxifloxacin had more treatment-associated side effects than improved treatment outcomes, and hence the authors did not recommend it for moxifloxacin-resistant cases [32]. Hence, more clarity is required on the use of fluoroquinolones and correlations to final treatment outcomes, particularly when resistance is detected.

The limitations of this study include the lack of comparison with liquid culture-DST data, the incompleteness of the data in the Ni-kshay database for a few parameters, and the lack of information of the drugs given to patients and/or the replacement drugs used for these patients. In addition, over the years, the drug regimen changed, leading to the comparison of multiple regimens across the study period and the lack of any significant correlations with outcomes. No statistically significant trend over time was observed, which is understandable given the routine use of FQs for any other bacterial infections [33,34,35].

## 5. Conclusions

In conclusion, this retrospective analysis reiterated that SL-LPA testing with MTBDR*sl* should be recommended for early detection of FQ resistance. The present study identified D94G (MUT3C) in *gyrA* and a1401g in *rrs* as the predominant mutation profile among FQ and SLID resistance, respectively. The mutations were corelated with levofloxacin or moxifloxacin resistance status and this should guide the choice of treatment regimen and the appropriate replacement of FQ drugs where required. A favorable treatment outcome among SL-DR patients was obtained in 37.8% of study subjects. The study recommends the monitoring of treatment outcomes for SL-DR patients, for better clinical management of SL-DR patients. Additionally, the patients not responding to treatment could be tested using NGS (either WGS or tNGS) at the National Reference Laboratories, to understand their comprehensive mutation profile faster than conventional phenotypic DST, to guide the treatment for a better outcome for the patient. With the availability of Indian and global catalogues of mutations, the associations of mutations with drug resistance are well defined, and better clinical correlation is expected when treatment outcomes are compared.

## Figures and Tables

**Table 1:** Proportion of second-line drug resistance in the study period, *n* (%).

Year	Second-Line Resistant Patients	Fluoroquinolone Resistant (FQ-R) Patients	Second-Line Injectable Drug Resistant (SLID-R) Patients	FQ + SLID Resistant Patients	Low Level Kan Resistant Patients *
2018	44	33 (75)	5 (11.4)	6 (13.6)	3/5 (60)
2019	85	54 (63.5)	19 (22.4)	12 (14.1)	2/19 (10.5)
2020	88	71 (80.7)	10 (11.4)	7 (8)	2/10 (20)
Total	217	158 (72.8)	34 (15.7)	25 (11.5)	7/34 (20.6)
*p*-Value	NA	0.263	0.660	0.284	0.251

Proportion of second-line drug resistance given as *n* along with % within brackets. A test of the trend of increasing/decreasing resistance across the year was performed. * Subset of SLI-resistant samples.

**Table 2:** Distribution of resistance mutations in second-line LPA-resistant isolates.

Mutations in Fluoroquinolone-Resistant Isolates (*n* = 183)							
**Gene**	**Band**	**Mutation**	**Year**			**Total**	***p*-Value**
			2018	2019	2020	(*n* = 183)	
			(*n* = 39)	(*n* = 66)	(*n* = 78)		
*gyrA*	MUT1(+)	A90V	3 (7.7)	10 (15.2)	11 (14.1)	24 (13.1)	0.402
	MUT2(+)	S91P	2 (5.1)	3 (4.5)	2 (2.6)	7 (3.8)	0.499
	MUT3A(+)	D94A	2 (5.1)	3 (4.5)	8 (10.3)	13 (7.1)	0.235
	MUT3B(+)	D94N/Y	2 (5.1)	6 (9.1)	5 (6.4)	13 (7.1)	0.894
	MUT3C(+)	D94G	19 (48.7)	23 (34.8)	33 (42.3)	75 (41)	0.832
	MUT3D(+)	D94H	0 (0)	0 (0)	0 (0)	0 (0)	NA
	WT1/2/3 (−)	codons 85–93/92–96	6 (15.4)	7 (10.6)	8 (10.3)	21 (11.5)	0.526
*gyrB*	MUT1(+)	N538D	0 (0)	0 (0)	1 (1.3)	1 (0.5)	0.333
	MUT2(+)	E540V	0 (0)	0 (0)	0 (0)	0 (0)	NA
	WT (−)	Codons 536–541	5 (12.8)	15 (22.7)	11 (14.1)	31 (16.9)	0.946
Mutations in second-line injectable-resistant isolates (*n* = 59)							
**Gene**	**Band**	**Mutation**	**Year**			**Total**	***p*-Value**
			2018	2019	2020	(*n* = 59)	
			(*n* = 11)	(*n* = 31)	(*n* = 17)		
*rrs*	MUT1(+)	a1401g	6 (54.5)	21 (67.7)	9 (52.9)	36 (61)	0.333
	MUT2(+)	g1484t	1 (9.1)	1 (3.2)	1 (5.9)	3 (5.1)	0.658
	WT1 (−)	1400/1484 region	1 (9.1)	7 (22.6)	5 (29.4)	13 (22)	0.628
*eis*	MUT1(+)	c-14t	1 (9.1)	1 (3.2)	1 (5.9)	3 (5.1)	0.658
	WT1/2/3 (−)	−37 to −2 region	2 (18.2)	2 (6.5)	1 (5.9)	5 (8.5)	0.260

Distribution of the mutation in *gyrA*, *gyrB*, *rrs,* and *eis* was consolidated as interpreted using GLI guidelines and is given in numbers along with % within brackets [3]. A test of the trend of increasing/decreasing resistance across the year was performed.

**Table 3:** Distribution of heteroresistance, inferred, and true resistance *n* (%).

**Gene**	**2018**	**2019**	**2020**	**Total**	***p*-Value**
	(*n* = 44)	(*n* = 85)	(*n* = 88)	(*n* = 217)	
True resistance					
*gyrA*	20 (45.5)	38 (44.7)	50 (56.8)	108 (49.8)	0.176
*gyrB*	0 (0)	0 (0)	0 (0)	0 (0)	NA
*rrs*	4 (9.1)	12 (14.1)	5 (5.7)	21 (9.7)	0.354
*eis*	1 (2.3)	0 (0)	0 (0)	1 (0.5)	0.136
Inferred resistance					
*gyrA*	6 (13.6)	7 (8.2)	8 (9.1)	21 (9.7)	0.526
*gyrB*	5 (11.4)	15 (17.6)	11 (12.5)	31 (14.3)	0.946
*rrs*	1 (2.3)	7 (8.2)	5 (5.7)	13 (6)	0.628
*eis*	2 (4.5)	2 (2.4)	1 (1.1)	5 (2.3)	0.260
Heteroresistance					
*gyrA*	8 (18.2)	6 (7.1)	8 (9.1)	22 (10.1)	0.217
*gyrB*	0 (0)	0 (0)	1 (1.1)	1 (0.5)	0.333
*rrs*	3 (6.8)	10 (11.8)	5 (5.7)	18 (8.3)	0.617
*eis*	0 (0)	1 (1.2)	1 (1.1)	2 (0.9)	0.600

Proportion of the three types of resistance (true, inferred, heteroresistance) across various genes are given in numbers, along with % within brackets.

**Table 4:** Treatment regimen given in the second-line drug-resistant patients, *n* (%).

**Treatment Regimen**	**2018**	**2019**	**2020**	**Total**
	(*n* = 44)	(*n* = 85)	(*n* = 88)	(*n* = 217)
All-oral longer regimen (MDR+/FQ or SLI)	0 (0)	9 (10.6)	36 (40.9)	45 (20.7)
Conventional MDR-TB regimen	4 (9.1)	2 (2.4)	3 (3.4)	9 (4.1)
Regimen for H Mono Poly	1 (2.3)	9 (10.6)	16 (18.2)	26 (12)
Regimen for MDR/RR + resistance to FQ/SLI	1 (2.3)	2 (2.4)	0 (0)	3 (1.4)
Regimen for XDR-TB	1 (2.3)	2 (2.4)	1 (1.1)	4 (1.8)
Regimen for newer drug for XDR-TB/failure for MDR/XDR-TB	6 (13.6)	18 (21.2)	3 (3.4)	27 (12.4)
Shorter MDR-TB regimen	5 (11.4)	12 (14.1)	7 (8)	24 (11.1)
Treatment details not available	26 (59.1)	31 (36.5)	22 (25)	79 (36.4)
*p* < 0.0001				

The treatment regimens of the patients who were resistant to either or both FQ/SLID during the study period were collected from the Ni-kshay database. The episodes of treatment post-declaration of SL-DST results were collected. Fisher’s exact test was used.

**Table 5:** Treatment outcome in the second-line-resistant patients, *n* (%).

**Treatment Outcome**	**2018**	**2019**	**2020**	**Total**
	(*n* = 44)	(*n* = 85)	(*n* = 88)	(*n* = 217)
Cured	6 (13.6)	19 (22.4)	17 (19.3)	42 (19.4)
Diagnosed not on treatment	0 (0)	0 (0)	3 (3.4)	3 (1.4)
Died	6 (13.6)	13 (15.3)	11 (12.5)	30 (13.8)
Lost to follow up	4 (9.1)	15 (17.6)	12 (13.6)	31 (14.3)
Patient refused	2 (4.5)	1 (1.2)	3 (3.4)	6 (2.8)
Treatment complete	6 (13.6)	14 (16.5)	20 (22.7)	40 (18.4)
Treatment failure	0 (0)	1 (1.2)	6 (6.8)	7 (3.2)
Treatment—regimen changed	5 (11.4)	4 (4.7)	4 (4.5)	13 (6)
Outcome details not available	15 (34.1)	18 (21.2)	12 (13.6)	45 (20.7)
*p* = 0.0704				

The treatment outcome of FQ and/or SLID-resistant patients were retrieved from the Ni-kshay database. Fisher’s exact test was used.

## Data Availability

Data available on request due to restrictions.
